# Effects of Nitrate Supplementation on Cardiovascular and Autonomic Reactivity in African-American Females

**DOI:** 10.1155/2014/676235

**Published:** 2014-02-23

**Authors:** Vernon Bond, Bryan H. Curry, R. George Adams, M. Sadegh Asadi, Kimani A. Stancil, Richard M. Millis, Georges E. Haddad

**Affiliations:** 1 Department of Health, Human Performance and Leisure Studies, and the Cancer Center Physical Medicine and Nutrition Laboratory, Howard University, Washington, DC 20059, USA; 2 Division of Cardiology, Department of Internal Medicine, Howard University College of Medicine and Howard University Hospital, Washington, DC 20060, USA; 3 Department of Neurology, Howard University College of Medicine and Howard University Hospital, Washington, DC 20060, USA; 4 Department of Physics and Astronomy, Howard University, Washington, DC 20059, USA; 5 Department of Physiology & Biophysics, Howard University College of Medicine, Washington, DC 20059, USA

## Abstract

Previous studies have shown that beetroot juice (BJ) decreases systolic blood pressure (SBP) and oxygen demand. This study tests the hypothesis that a beetroot juice (BJ) treatment increases heart rate variability (HRV) measured by the average standard deviation of normal-normal electrocardiogram RR intervals (SDNN) and the low frequency (LF), mainly sympathetic, fast Fourier transform spectral index of HRV. The subjects were 13 healthy young adult African-American females. Placebo control orange juice (OJ) and BJ treatments were given on separate days. Blood nitric oxide [NO], SBP and RR intervals were measured at rest and at constant workloads set to 40% and 80% of the predetermined VO_2peak_. Two hours after ingestion the BJ treatment increased [NO] and decreased SBP. BJ also increased SDNN at rest and at the 40% VO_2peak_ workload, without significant effects on LF. SDNN was significantly greater after the BJ than after the OJ treatment, across the two physical activity conditions and SDNN was (negatively) correlated with SBP. These results suggest that BJ decreases SBP and increases HRV at rest and during aerobic exercise. Similar results in subjects with prehypertension or hypertension could translate to a dietary nitrate treatment for hypertension.

## 1. Introduction

Studies have consistently reported a greater prevalence of hypertension in African Americans than in other ethnic groups [1]. Adolescent and young adult African Americans consistently have exhibited greater pressor responses to laboratory mental and physical stressors than Caucasian Americans [2–8]. Because it characterizes the early stages of hypertension, it is speculated that such heightened pressor responsiveness to stress may be a key factor in the pathogenesis of hypertension [9–11]. Blood pressure reactivity during laboratory stressors has also been found to be more predictive of increased ambulatory blood pressure measured three years later, in African Americans compared to Caucasians [12]. In examining blood pressure responses to the physical stress of cycle ergometry in normotensive individuals, 82% of blood pressure hyperresponders developed hypertension after a two-year follow-up [13].

Nitric oxide (NO) is a potent endogenous vasodilator [14] that plays an important role in regulating blood pressure. An ethnicity-specific difference in endothelial NO with less NO production in blacks than whites is reported [15]. There is substantial evidence that the inorganic nitrate anion, either generated *in vivo* as an oxidative metabolite of NO itself [16] or ingested through the diet, main dietary sources being green leafy vegetables [17], might provide endogenous sources of NO independent of the conventional NO synthase pathway [18, 19]. Several studies on healthy young subjects have demonstrated increases in plasma nitrite above placebo of approximately 100–300 nM ([20–25] following various administrations of inorganic nitrate, including chronic (4–6 days) [20–22, 24] and acute (>4 hours) [24,25] consumption of beetroot juice. Therefore, this study was designed to test the hypothesis that acute dietary nitrate supplementation using beetroot juice would decrease the sympathetic autonomic influences on heart rate, blood pressure, hemodynamic, and autonomic responsiveness to the physical stress of aerobic exercise in healthy young adult African Americans.

## 2. Methods

### 2.1. Subjects

The subjects were 13 healthy normotensive young adult African American females who were physically active but not exercise-trained. None of the subjects were smokers or drinkers and free of any medication intake (birth control, etc.). The procedures outlined in the study were approved by the Howard University Institutional Human Participants Review Board. Following explaining the study procedures and risks, all subjects gave their written informed consent before commencement of the study. Subjects were instructed to arrive at the laboratory in a rested state, at least three hours postprandial, and to avoid strenuous exercise in the 24 hours preceding each testing session. All tests were performed at the similar time of day.

### 2.2. Procedures

The study design consisted of the subjects reporting to the laboratory on three occasions, over a 3–7 week period. The first laboratory visit consisted of the subjects performing a progressive exercise test determining peak oxygen uptake (VO_2peak_). The progressive exercise test of VO_2peak_ was performed on an electronically braked cycle leg cycle ergometer (Lobe Corival, Groningen, The Netherlands). The subject performed a three-minute warmup of cycling with no workload, after which the exercise intensity was increased at a rate of 20 W every three minutes until volitional fatigue. The metabolic measure of VO_2peak_ was defined by the VO_2_ value generated during the last minute of the progressive exercise test. Respiratory measures of expired O_2_, carbon dioxide, and minute-ventilation were made during the progressive exercise test using a Physio-Dyne Max-II metabolic system (Physio-Dyne Instrument Corp., Quogue, NY). Additionally, in the first visit, the subject’s body composition was measured by the dual energy X-ray absorptiometry method using an Hologic QDR whole body scanner (Waltham, MA).

After measuring VO_2peak_ in the first laboratory visit, the subjects were randomly assigned to two visits in which they consumed 500 mL of either nitrate rich beetroot juice (Biotta Inc, Carmel, IN) or placebo/control (orange juice) and performed a standardized exercise test on the cycle ergometer. We selected orange juice as the control because it is matched with beetroot juice for calories (200 Cal), carbohydrates (52 g), fat (negligible), and protein (4 g) content and is also similar in texture and is high in antioxidants [26]. Biotta beetroot juice has a listed content of nitrate of 1500 mg/L. The subject was instructed to ingest the beverage over 20 min and left for 120 min in order to digest and process the beverage [18, 24, 25]. To avoid any possible interference with the processing of nitrate or nitrite in the saliva or stomach, the subject was instructed to refrain from using antibacterial mouthwash products. There were no issues or side effects observed for the beetroot juice or control supplementations. An observation of red urine and red stool was reported by the subjects. In laboratory visits two and three, 120 min after ingesting the beetroot juice and control supplements, the subjects performed an exercise test working at defined levels of low and high physical stress. After a 5 min baseline period, the subject exercise at a relative work intensity of 40% and 80% of each subject’s predetermined VO_2peak_, defining low and high levels of physical stress, respectively. Both bouts of submaximal exercise were of 5 min duration. Before the submaximal work bouts, blood pressure, heart rate, and cardiac output were measured. Additionally, venous blood was collected for determination of NO. To limit the confounding influences of menstrual hormonal changes on blood pressure, the females were tested during the luteal phase of their menstrual cycle. Before the exercise bout, the NO concentration in venous blood, blood pressure (BP), heart rate (HR), cardiac output (CO), total peripheral vascular resistance (TPR), and electrocardiogram heart rate variability (HRV) measurements were performed.

### 2.3. Measurements

A blood sample (100 *μ*L) was collected into a tube containing EDTA. To deproteinize the blood, the sample was placed in 300 *μ*L of 20% trichloroacetic acid and centrifuged at 15,000 g for three minutes. Then, 10 *μ*L of a nitrite preservation solution containing 0.8 M ferricyanide, 10 mM-N-ethylmaleimide, and 1% NP-40 was added to the mixture. NO was measured (within 20 minutes of collection) by the amperometric method using an NO electrode system (Innovative Instruments, Inc., Tampa, FL). The electrode was calibrated using a standard nitrite concentration of 50 and 100 nM. The NO recording system has an analog to digital resolution of 24 bit allowing resolution of currents down to 0.1 pA. Assay measurement of NO was performed using the average of duplicate values.

Blood pressure of the brachial artery was measured by a SunTech 4240 (SunTech, Medical Instruments, Raleigh, NC) automated monitor using R-wave gating and k-sounds for measuring absolute systolic and diastolic blood pressure. Mean arterial pressure (MAP) was estimated by the formula MAP = diastolic blood pressure plus 1/3 pulse pressure. To verify the automated device blood pressure readings, we connected the monitor to a mercury column using a Y-tube connector and assessed the Korotkoff sounds using an audio headphone.

Heart rate and estimate of CO measurements were made using the SORBA model CIC-1000 impedance electrocardiography system, software version 7.2 (SORBA Medical Systems, Inc., Brookfield, WI). The subjects were prepared with four electrodes to measure impedance. The first electrode was placed on the forehead. The second electrode was placed at the base of the neck on the left side, the third at the midaxillary line at the level of the xiphoid process, and the fourth at the midaxillary line at the region of the greater trochanter. Two electrocardiogram (ECG) electrodes were placed on the subject’s right and left arm, respectively. The SORBA system continuously sampled signals for CO using the Kubicek equation [27] to calculate stroke volume. Total peripheral vascular resistance (TPR) was calculated from the formula TPR (dyne·sec·cm^−5^) = 80 × MAP (mm Hg)/CO (L·min^−1^).

The effects of beetroot juice on respiratory sinus arrhythmia and autonomic signaling were measured by the average standard deviation of the normal-normal RR intervals (SDNN) and fast Fourier transform analysis (FFT) of the RR intervals over 5 min during rest and exercise at constant workloads set to 40% and 80% of each subject’s predetermined VO_2peak_, using specialized HRV software (Nevrokard, Slovenia).

### 2.4. Data Analysis

For statistical analysis paired (two-tailed) Student’s *t*-tests were used to determine significance of treatment-related differences in cardiac output (CO), heart rate (HR), systolic and diastolic blood pressure (SBP, DBP), heart rate × systolic blood pressure product (RPP), total peripheral resistance (TPR), average standard deviation of the normal-normal electrocardiogram RR intervals, and the low and high frequency (LF, HF) FFT spectral components of HRV. Linear regression analysis and Pearson’s product-moment correlation coefficient (*r*) were used to determine the significance of correlations across the control and beetroot juice treatments and the physical activity conditions, as well as across the physical activity conditions for each of the treatments. Differences were considered to be significant at *P* < 0.05.

## 3. Results

Table 1 summarizes the physical characteristics of the study subjects. Values for the percentage of body fat and VO_2peak_ indicate that the subjects were overweight and exhibited a low level of cardiorespiratory fitness.

**Figure 1:**
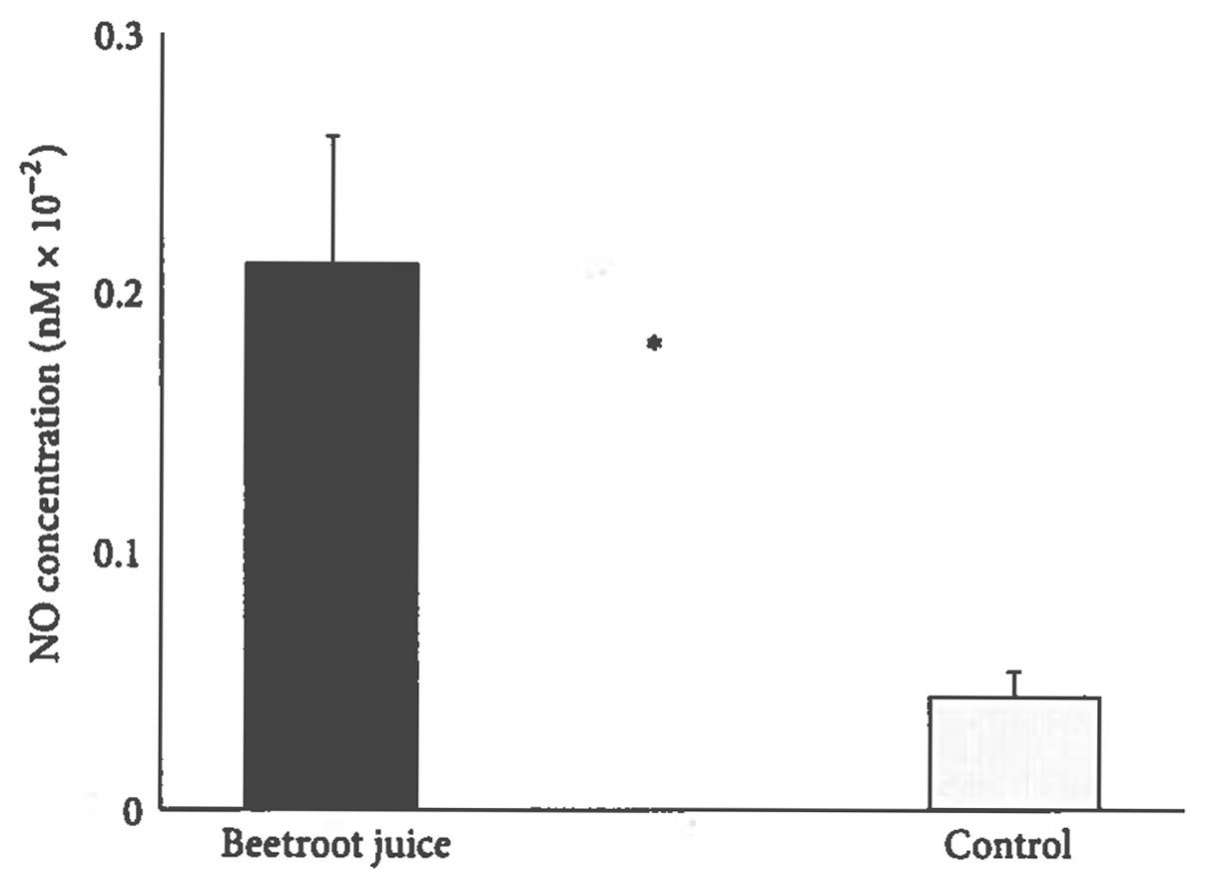
Effects of beetroot juice treatment on blood nitric oxide level. Bars represent mean ± standard error measurements of blood nitric oxide (NO) concentration, expressed in nM units. The subjects were 13 healthy young adult females administered a placebo control orange juice and an isocaloric beetroot juice treatment on separate days. Asterisk (*) indicates a significant treatment-related difference at *P* < 0.0001.

Figure 1 shows that the blood NO concentration increased significantly 120 min after supplementation with nitrate-rich beetroot juice (21.1 ± 4.9 nM versus 4.4 ± 1.0 nM, *P* < 0.0001).

**Figure 2:**
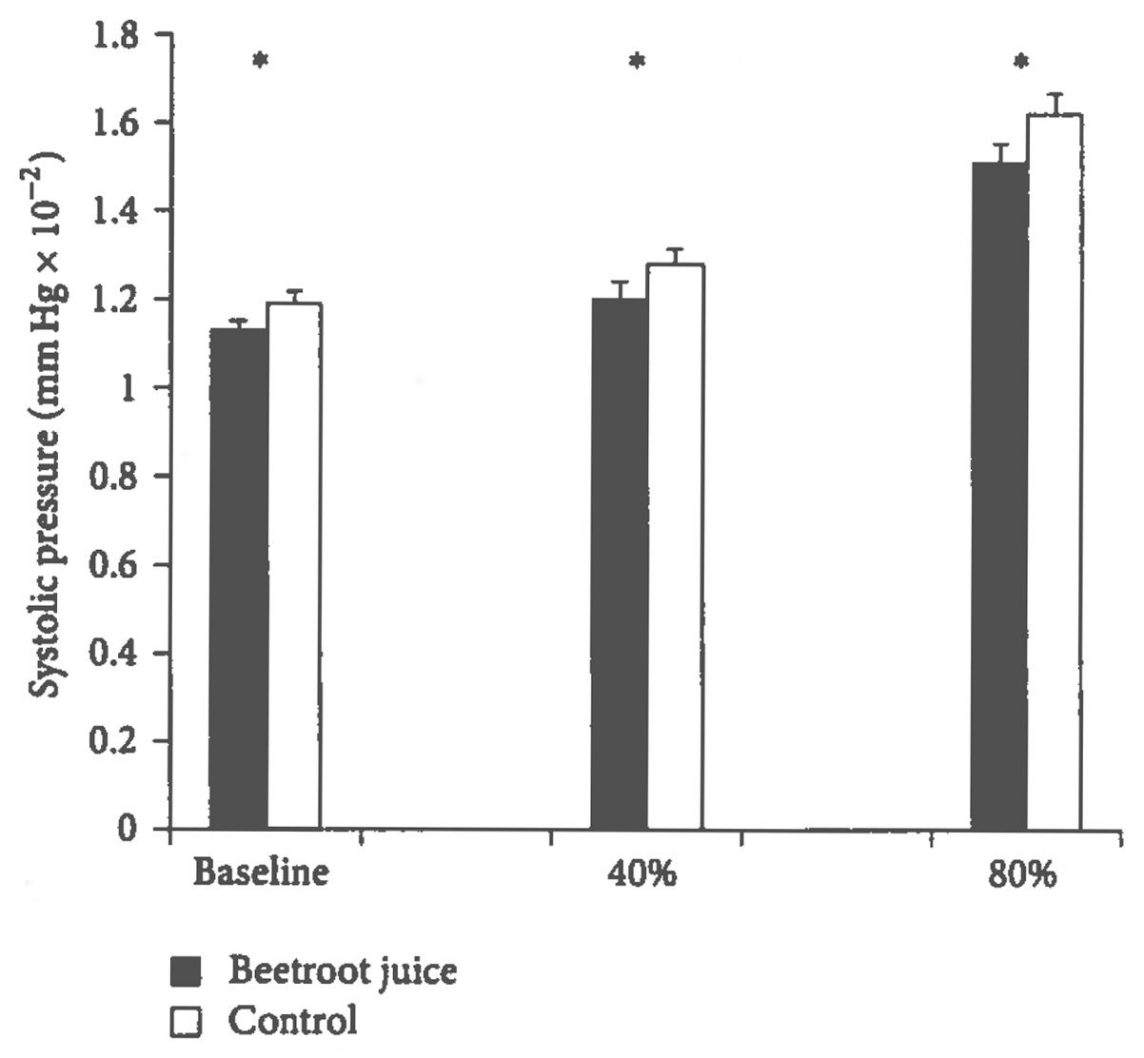
Effects of beetroot juice treatment on systolic blood pressure. Bars represent mean ± standard error measurements of systolic blood pressure, expressed in mm Hg, performed at rest (baseline) at constant aerobic exercise workloads set to 40% and 80% of the predetermined peak oxygen consumption (VO_2peak_). The subjects were 13 healthy young adult females administered a placebo control orange juice and an isocaloric beetroot juice treatment on separate days. Asterisk (*) indicates a significant treatment-related difference at *P* < 0.05.

Figure 2 presents the blood pressure data demonstrating that, after the nitrate-rich beetroot juice treatment, SBP was significantly lower than control during the baseline resting condition (113.8 ± 2.1 versus 119.7 ± 2.8 mm Hg, *P* < 0.05), 40% VO_2peak_ (128.7 ± 3.3 versus 120.6 ± 4.1 mm Hg, *P* < 0.05), and 80% VO_2peak_ (162.6 ± 4.6 versus 151.6 ± 4.3 mm Hg; *P* < 0.05). DBP tended to be reduced following nitrate-rich beetroot juice at control baseline, 40% VO_2peak_, and 80% VO_2peak_ workloads, but the differences were not significant. Across the baseline resting and two exercise conditions, SBP was found to be significantly lower after the beetroot juice than after the control orange juice treatment (128.3 ± 3.6 versus 137.1 ± 3.9 mm Hg, *P* < 0.0001).

Table 2 illustrates the cardiovascular hemodynamic measures for HR, CO, and TPR at baseline and during the low and high levels of physical stress. Nitrate-rich beetroot juice did not significantly alter HR or CO during the baseline rest condition, as well as at the two levels of exercise stress. TPR was computed to be significantly lower during rest and during exercise at the 40% VO_2peak_ and 80% VO_2peak_ workloads (*P* < 0.05). The beetroot juice treatment-related decrements in SBP and TPR were associated with parallel, significant decrements in the heart rate-systolic pressure product (RPP). Across the three conditions, RPP was significantly lower after the beetroot juice than after the control treatment (14,883 ± 1,060 versus 16,157 ± 1,130 beats·mm Hg·min^−1^, *P* < 0.0001),.

**Figure 3:**
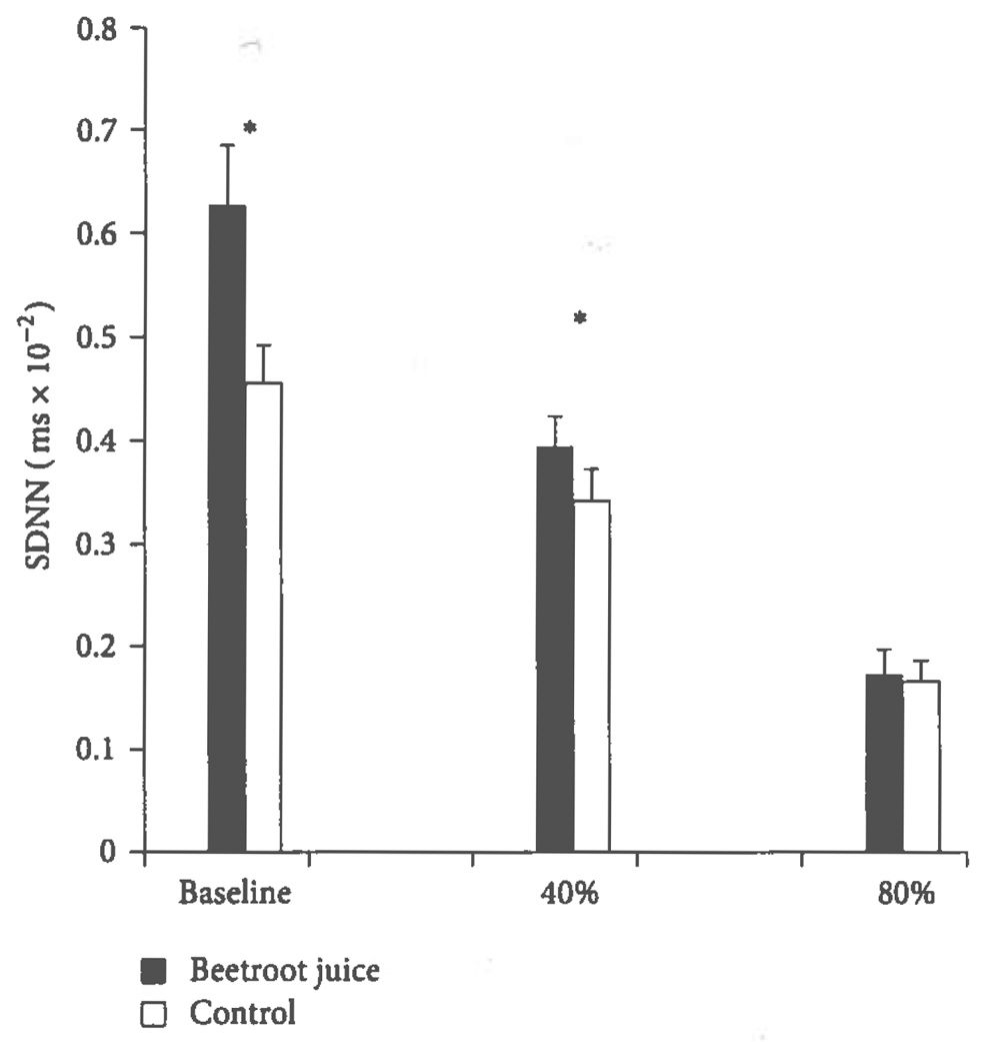
Effects of beetroot juice treatment on heart rate variability. Bars represent mean ± standard error measurements of the average electrocardiogram RR (interbeat) interval, expressed in ms, performed at rest (baseline) at constant aerobic exercise workloads set to 40% and 80% of the predetermined peak oxygen consumption (VO_2peak_). The subjects were 13 healthy young adult females administered a placebo control orange juice and an isocaloric beetroot juice treatment on separate days. Asterisk (*) indicates a significant treatment-related difference at *P* < 0.05.

Figure 3 shows significantly larger SDNN, after the beetroot juice treatment at rest and during the 40% VO_2peak_ workload. Across the three physical activity conditions, SDNN was significantly higher and HR, SBP, and RPP were significantly lower after the beetroot juice than after the control orange juice treatment (mean ± standard error SDNN: 41.4 ± 4.0 ms versus 33.5 ± 2.7 ms, *P* < 0.0005; HR: 112.3 ± 5.3 versus 114.1 ± 5.2, *P* = 0.03; SBP: 128.3 ± 3.6 mm Hg versus 137.1 ± 3.9 mm Hg, *P* < 0.0005; RPP: 14,882.3 ± 1060.4 mm Hg·beats·min^−1^ versus 16,156.7 ± 1129.6 mm Hg·beats·min^−1^, *P* < 0.0005). SDNN was significantly correlated with HR (*r* = −0.81, *P* < 0.0001), with SBP (*r* = −0.53, *P* < 0.0001), and with RPP (*r* = −0.75, *P* < 0.0001) across both treatments (orange juice and beetroot juice) and the three conditions (rest, 40% VO_2peak_ and 80% VO_2peak_ workloads).

Table 3 presents the results of FFT spectral analysis of autonomic signaling demonstrating that the treatment-related differences in LF, HF, and LF/HF spectral measurements of HRV were not significant.

## 4. Discussion

The main finding of this study is that beetroot juice increases SDNN. SDNN is a time-domain HRV measure of the autonomic (sympathovagal) influence on HR and was found to be inversely correlated with both SBP and with RPP, an index of myocardial oxygen demand. The significance of larger SDNN associated with the beetroot juice than with the placebo orange juice treatment is demonstrated by other studies wherein SDNN was positively correlated with the LF/HF spectral power ratio. Generally, larger LF/HF indicates greater sympathetic neural signaling, whereas smaller LF/HF indicates greater parasympathetic (vagal) autonomic signaling [28]. Our measurements of LF and HF failed to show significant treatment-related differences likely because of insensitivity of such measurements during aerobic exercise [29] and the LF/HF raising effect of high-carbohydrate beverages associated with high respiratory quotient [30].

Increased SDNN without a change in LF/HF has been reported following administration of simvastatin to animals subjected to experimental heart failure [31]. Thus, under some conditions, LF/HF may not be as sensitive as SDNN as an HRV indicator of more vagal and less sympathetic signaling. There are also conditions in which changes in LF/HF are not associated with changes in SDNN [28], but the reasons have not been fully investigated.

In the present study, the larger SDNN associated with the beetroot juice treatment occurred in the absence of treatment-related differences in HR but in the presence of treatment-related decrements in RPP and TPR. These effects were also associated with significant negative (inverse) correlations between SDNN and these variables across the two treatment conditions. Increases in SBP and RPP are common correlates of high sympathetic activity [32]. Thus, the beetroot juice treatment-related differences (increases) in SDNN in this study appear to be indicative of increased RSA, more vagal and less sympathetic signaling to the heart. Larger SDNN was found at rest and during submaximal exercise where the larger SDNN translated into a smaller physiological decrement in SDNN at the 40% VO_2peak_ workload. A beetroot juice treatment-related difference in SDNN was not observed at the 80% VO_2peak_ workload, likely because of insensitivity of the measurement at very high heart rates.

There is some evidence from prior studies, in experimental animals and in humans, that the dietary nitrate-induced increase in plasma NO played a key role in the beetroot juice treatment-related increases in SDNN. Decreased SDNN has been observed following administration of an inhibitor of neuronally released NO in anesthetized dogs [33] and high plasma concentration of endothelin-1, an endogenous mediator of hypertension, is shown to be correlated with low plasma NO levels and low LF/HF in patients with slow coronary artery flow [34]. These reports suggest that decreased bioavailability of NO, by any means, might decrease HRV and worsen sympathovagal signaling. On the other hand, sildenafil, a drug that mimics NO by increasing intracellular levels of cGMP, is reported to decrease LF/HF, an indication of more vagal, less sympathetic influence on HR [35]. This finding suggests the potential for dissociation of NO and cGMP in the intracellular transduction pathway signaling of cardiac myocytes. Dietary administration of L-arginine, the main precursor of NO, is reported to increase the urine concentrations of NO and cGMP and of LF/HF in healthy young adults [36] suggesting that, under some circumstances, high blood NO is associated with increased sympathetic signaling. On the other hand, treatment of stable heart failure patients with the NO donor glyceryl trinitrate failed to increase LF/HF [37], an effect which could have been an indication of either dose-effect or health/disease dependency. These effects of NO appear to be what might be expected when blood NO is increased by oxidative stress.

There are huge stores of nitrate, and nitrite and nitrothiol in cutaneous tissues which function as NO donor molecules. The primary sources of endogenous NO are endothelial, inducible, and neuronal NO synthase, eNOs, iNOS, and nNOS, respectively, and there is substantial evidence that structural and functional changes in these NOS isoforms may modulate HRV measurements. For example, in patients with specific NOS polymorphisms, greater LF/HF is reported in CHF patients [38], as well as paradoxical LF/HF-lowering responses to exercise training in healthy humans [39]. Moreover, treatment of mice with caveolin-1, an endogenous activator of eNOS, is shown to increase the sympathetic influence on blood pressure variability [40].

Over the past twenty years, a large database has emerged concerning the effects of inhibitors of NO synthase (NOS) demonstrating that increased bioavailability of NO plays a significant role in the mechanisms related to sympathetic-parasympathetic imbalances. For example, the NOS inhibitor L-NAME is shown to increase the predominance of sympathetic measures of blood pressure variability and baroreceptor reflex sensitivity and higher plasma NO levels are reported in heart failure patients than in control subjects [41]. L-NAME treatment is shown to block the exercise-induced decrement in LF/HF in normal rats [42] and the L-NAME experimental rat model of hypertension is associated with low LF/HF and high plasma levels of malondialdehyde (MDA), a biochemical marker of oxidative stress [43]. Furthermore, after chemical (6-hydroxydopamine) sympathectomy, MDA decreased, with increased LF/HF [43]. Additionally, L-NAME treatment and NO inhibition in the hypothalamic paraventricular nucleus are reported to decrease the physiological increment in LF/HF normally observed during exercise in swim-trained rats but had no effect in sedentary, untrained rats [44].

## 5. Conclusion

In summary, the results of this study demonstrate that a single beetroot juice treatment may produce significantly larger SDNN at rest and smaller exercise-induced decrement in SDNN, associated with a markedly increased plasma NO concentration in healthy humans. These findings support previous reports that dietary nitrate supplementation with beetroot juice has the potential to decrease blood pressure, vascular resistance, and myocardial oxygen demand in both resting and exercising subjects. The increases in SDNN appear to be indicative of less sympathetic influence on heart rate which might be dependent on NO bioavailability. However, these results should be interpreted cautiously pending verification by more direct measures of sympathetic signaling to the arterial vasculature such as baroreceptor sensitivity and direct microneurography. Because high blood pressure, vascular resistance, myocardial oxygen demand are positively correlated with the severity of cardiovascular disease [32], beetroot juice and other dietary sources of nitrates may translate into an effective prevention and treatment strategy for cardiovascular disease which may serve to reduce the thermodynamic effects of sympathetic overactivity.

## Figures and Tables

**Table 1:** Physical characteristics of study subjects (*N* = 13).

Variable	Values
Age (yr)	20.9 ± 0.3
Height (cm)	161.8 ± 2.1
Weight (kg)	61.4 ± 2.2
Body fat (%)	30.7 ± 2.5
VO_2peak_ (ml·kg^−1^·min^−1^)	27.8 ± 1.7
HR_peak_ (b·min^−1^)	177.5 ± 2.6
HR_rest_ (b·min^−1^)	87.8 ± 2.8
SBP_rest_ (mm Hg)	116.2 ± 3.2
DBP_rest_ (mm Hg)	82.5 ± 2.7

Values are means ± standard errors.

VO_2peak_: peak oxygen uptake; HR_peak_: heart rate peak; HR_rest_: heart rate rest; SBP_rest_: systolic blood pressure rest; DBP_rest_: diastolic blood pressure rest.

**Table 2:** Cardiovascular hemodynamic responses at baseline and during submaximal exercise with nitrate-rich beetroot juice compared to control (*N* = 13).

Variable	Beetroot juice	Control
Heart rate (b·min^−1^)	81.5 ± 2.8	86.0 ± 3.2
CO (L·min^−1^)	5.0 ± 0.1	4.8 ± 0.1
TPR (dyne·sec·cm^−5^)	1545 ± 66.5	1670.8 ± 92*

40% VO_2peak_		
Heart rate (b·min^−1^)	102.3 ± 2.8	104 ± 3.1
CO (L·min^−1^)	6.8 ± 0.2	6.7 ± 0.3
TPR (dyne·sec·cm^−5^)	1083.6 ± 62.2	1171.5 ± 75.7*

80% VO_2peak_		
Heart rate (b·min^−1^)	153.9 ± 3.0	153.7 ± 2.8
CO	11.6 ± 0.6	11.5 ± 0.5
TPR (dyne·sec·cm^−5^)	700.5 ± 48.6	747.0 ± 48.8*

Values are means ± standard errors.

* Significant difference between beetroot juice and control treatment at *P* < 0.05, CO: cardiac output; TPR: total peripheral resistance.

**Table 3:** Cardiac autonomic responses at baseline fand during submaximal exercise with nitrate-rich beetroot juice compared to control (*N* = 13).

Variable	Beetroot juice	Control
HF (nu)	48.5 ± 5.1	40.2 ± 5.0
LF (nu)	66.7 ± 8.0	73.6 ± 7.4
LF/HF	2.4 ± 0.4	1.7 ± 0.3

40% VO_2peak_		
HF (nu)	30.8 ± 3.0	29.1 ± 4.5
LF (nu)	99.8 ± 9.3	103 ± 13.1
LF/HF	5.3 ± 1.1	3.6 ± 0.6

80% VO_2peak_		
HF (nu)	10.0 ± 0.9	10.0 ± 1.8
LF(nu)	99.0 ± 22.4	114.5 ± 21.6
LF/HF	11.5 ± 2.6	10.6 ± 1.6

Values are means ± standard errors. HF: high frequency; LF: low frequency; nu: normalized unit.
